# Effect of astragaloside on diaphragm cell apoptosis in chronic obstructive pulmonary disease

**DOI:** 10.1002/fsn3.1751

**Published:** 2020-11-18

**Authors:** Li Wang, Yan Tan, Lin Gao, Jing Lei, Chen Chen, Yi Shi

**Affiliations:** ^1^ Department of Respiratory and Critical Medicine Nanjing First Hospital Nanjing Medical University Nanjing China; ^2^ Department of Respiratory and Critical Medicine Jinling Hospital Nanjing Medical University Nanjing China

**Keywords:** apoptosis, astragaloside, caspase‐3, caspase‐9, chronic obstructive pulmonary disease, diaphragm, p‐AKT

## Abstract

**Purpose:**

This study aimed to discuss the effects and relative mechanisms of astragaloside (AST) on diaphragm cell apoptosis in mice with chronic obstructive pulmonary disease (COPD).

**Materials and methods:**

The mouse models of COPD were established by passive smoking. The pathological changes in lung and diaphragm tissues were observed by hematoxylin and eosin staining and evaluating the number of apoptotic cells of the diaphragm via a terminal deoxynucleotidyl transferase dUTP nick‐end labeling assay. The relative protein expression levels of AKT, p‐AKT, caspase‐3, and caspase‐9 were measured through immunohistochemistry and Western blot assay.

**Results:**

In comparison with the normal control mice, the pathological change and number of apoptotic cells deteriorated in the lung and diaphragm tissues of COPD model mice. With AST supplement, the pathological change and the number of apoptotic cells significantly improved (*p* < .05). With AKT inhibitor intervention, the effects of AST treatment disappeared. p‐AKT, caspase‐3, and caspase‐9 protein expression was stimulated in the model group but was depressed in the AST‐treated groups.

**Conclusion:**

Our in vivo study revealed that AST improved COPD‐induced diaphragm apoptosis by regulating and depressing AKT activities.

## Graphical abstract

This work clearly discusses the effects and mechanisms of astragaloside in COPD.

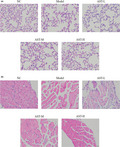

## 1 INTRODUCTION

Respiratory muscle fatigue is the most important condition causing respiratory failure induced by chronic obstructive pulmonary disease (COPD). As the primary muscle for breathing, the respiratory diaphragm contributes to 80% of the respiratory volume at rest during muscle contraction. Therefore, diaphragmatic fatigue is a pathophysiological determinant of respiratory failure. Reportedly, the excessive apoptosis of diaphragm cells is closely related to the development and progression of COPD (Degens et al., 2007).

Astragaloside (AST), a key active compound found in *Astragalus membranaceus*, helps promote vasodilation, prevents endothelial dysfunction, and improves cardiac energy metabolism, inflammatory responses, and antioxidation (Huang et al., 2016). It is an effective ingredient in treating injuries induced by pulmonary diseases, such as pulmonary fibrosis and pneumonia (Chen et al., 2016; Li et al., 2017; Qian, Cai, Qian, Zhang, & Wang, 2018). However, current research on the effect of AST on diaphragm cells of patients with COPD is relatively limited.

In this study, several COPD mouse models were established, and mice were treated with AST injections. Pathological changes in their pulmonary tissues, diaphragm cells, and apoptotic cells in the diaphragm were closely observed. The expression levels of AKT, p‐AKT, caspase‐3, and caspase‐9 were assessed to investigate the effect of AST on diaphragm cell apoptosis in mice with COPD and offer a new approach for the clinical treatment of diaphragmatic fatigue.

## 2 MATERIALS AND METHODS

### 2.1 Materials

The following materials were used in this study: 36 normal female C57BL/6 mice (Shanghai SLAC Laboratory Animal Co. Ltd); H and E staining kit (NanJing KeyGen Biotech, China); terminal deoxynucleotidyl transferase dUTP nick‐end labeling (TUNEL) assay kit for apoptotic cell detection, primary antibodies, including AKT, p‐AKT, caspase‐3, and caspase‐9, and AKT inhibitor (Abcam); SP ELISA kit (Fuzhou Maixin Biotech); and AST (Aladdin; HPLC ≥98%).

### 2.2 Grouping and modeling

The 36 mice were divided into a normal control (NC) group, a COPD model (model) group, and four intervention groups by using a random number table. The three intervention groups included an AST‐L group receiving a lower dose of AST (10 mg/kg·d), an AST‐M group administered with a medium dose of AST (20 mg/kg·d), an AST‐H group treated with a higher dose of AST (40 mg/kg·d), and an AST + AKT inhibitor group subjected to the combined treatment of AST (40 mg/kg·d) and AKT inhibitor (30 mg/kg·d).

In the model group, the mice were fed adaptively for 1 week and exposed to ozone generator (Nanjing Wohuan Ozone Technology Co., Ltd.) for 3 hr. The ozone concentration was controlled at 2.5 ppm three times a week for 6 weeks.

In the NC group, the mice were treated with NC. AST‐L, AST‐M, AST‐H, and AST + AKT inhibitor groups. Injection was performed 30 min prior to each modeling. Afterward, a COPD model was taken by using an ozone generator. The experiment was performed for 6 weeks.

### 2.3 Pathological specimen preparation

After 6 weeks, the mice were anesthetized with 10% chloral hydrate for 30 min before an incision was made to expose the thoracic cavity and collect blood from the IVC. The right lung was removed, fixed with 10% neutral formaldehyde for 24 hr, and subjected to routine paraffin processing and hematoxylin and eosin (H and E) staining. The degree of emphysema was assessed in terms of the mean linear intercept and the mean alveolus number. The diaphragm was removed intact, and the left section was largely fixed with 10% neutral formaldehyde for immunohistochemistry (IHC) staining and TUNEL. The right section was divided into two parts: One was stored in liquid nitrogen and the other was used for Western blot (WB).

### 2.4 Pathological examination

Lung and diaphragm tissues were collected from the mice, washed twice with cold normal saline, and fixed with 4% paraformaldehyde. Paraffin sections were prepared routinely and dewaxed conventionally. H and E staining was performed, and pathological changes were observed under a digital microscope to capture microscopy images.

### 2.5 Apoptotic rate of diaphragm muscle cells

A TUNEL assay was performed to determine the apoptotic rate of diaphragm muscle cells. Each diaphragm muscle sample was used for the preparation of five microtome sections. After the specimens were subjected to TUNEL staining, positive cells were counted at 400× magnification in five different vision fields, and the TUNEL‐positive rate was determined.

### 2.6 IHC assay of AKT, p‐AKT, caspase‐3, and caspase‐9 expression in diaphragm tissues

After routine dewaxing and rehydration in a gradient ethanol solution were conducted, paraffin sections were subjected to microwave‐citrate antigen retrieval by using sodium citrate buffer (0.01 mol/L, pH 6.0) and then incubated with 3% H_2_O_2_ at room temperature for 20 min. Different primary antibodies, including AKT (1:50), p‐AKT (1:50), caspase‐3 (1:50), and caspase‐9 (1:50), were applied for overnight incubation. Afterward, the sections were washed with PBS and used for secondary antibody incubation (dilution ratio = 1:200) at 37°C for 30 min. The sections were washed with PBS, stained with a DAB reagent, counterstained with hematoxylin, and mounted with a colorless and transparent mounting medium. Microscopy images were analyzed using Image J.

### 2.7 Protein expression detection by WB

Lysis buffer (0.5 ml) was added and thoroughly mixed with 100 mg of the tissues cut into small pieces. After the mixture was allowed to stand for 10 min at 4°C, 1 ml of the extraction reagent was added and mixed well with the solution. Then, the resulting solution was left to stand for another 10 min at 4°C. Subsequently, it was centrifuged at 10,000 *g* at 4°C for 10 min to obtain two phases of liquid separated by a protein layer. The liquid was removed, and the proteins were dried at 4°C. Then, 2% SDS was used to dissolve the proteins, and the BCA assay was performed for protein quantification. The protein solution was fully mixed with a loading buffer at a ratio of 3:1 and then placed in a water bath at 95°C for 10 min (denaturation). Wells were loaded with 30 μg of proteins, and SDS‐PAGE was performed at 80 V for 2 hr. Membranes were transferred at 70 V for another 2 hr. After the membranes were mounted with 5% skim milk/TBST for 1 hr, the proteins were incubated overnight at 4°C with AKT, p‐AKT, caspase‐3, and caspase‐9 (1:500) and then placed onto a thermostatic shaking table for secondary antibody incubation (1:5,000) at room temperature for 1 hr. A BeyoECL Star Ultrasensitive ECL chemiluminescence kit (Beyotime) was used for development, and a Bio‐Rad gel imaging system was employed to capture images. Image analysis was performed using Image Lab 5.0.

### 2.8 Statistical Analysis

SPSS22.0 was used for data analysis. Data were expressed as mean ± *SD*. The normal distribution and homogeneity of variance were examined. One‐way ANOVA was conducted for intergroup comparison, and an LSD *t* test was conducted for the intergroup comparison of quantitative variables. Data with *p* < .05 were considered statistically significant.

## 3 RESULTS

### 3.1 Effect of AST on the pathological characteristics of the lung and diaphragm tissues of mice with COPD

**FIGURE 1:**
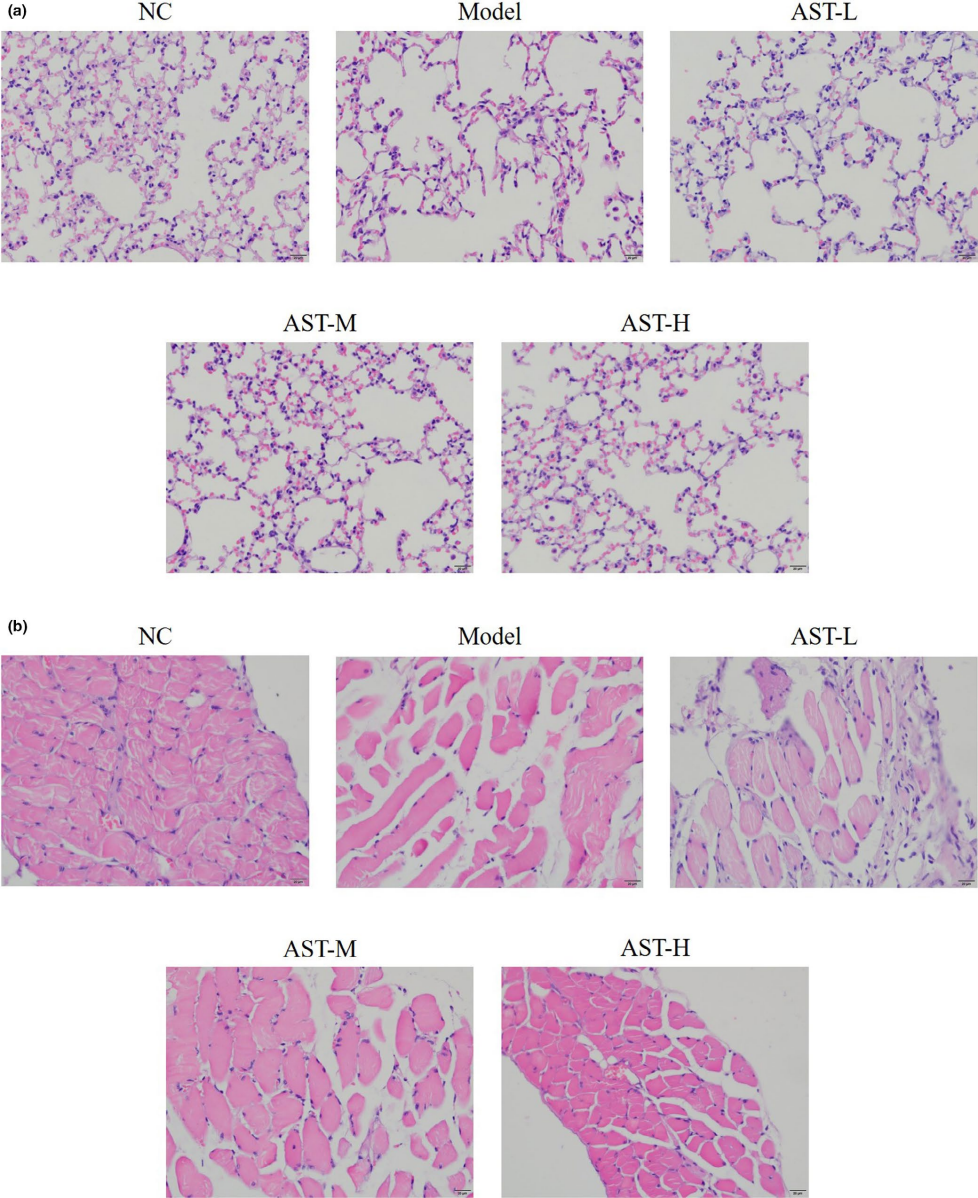
Pathological observations of the lung and diaphragm tissues via HE staining. NC, mice treated with normal control; model, COPD model mice; AST‐L, COPD model mice treated with low‐dose AST; AST‐M, COPD model mice treated with middle‐dose AST; AST‐H, COPD model mice treated with high‐dose AST. (a) Pathological observations of the lung tissues via HE staining (400×). (b) Pathological observations of the diaphragm via HE staining (400×)

HE staining revealed that alveolar spaces were significantly enlarged and alveolar walls were destroyed in the model group compared with those of the NC group. Nevertheless, the alveolar spaces became much smaller, and the destruction of the alveolar walls was considerably alleviated after the mice were treated with AST (Figure 1a). The pathological analysis of diaphragm tissues revealed severe diaphragmatic tears in the model group, but this condition was significantly improved after the AST treatment. Moreover, a dose–response relationship was observed between AST and the condition of diaphragmatic tears (Figure 1b). These results proved the effectiveness of the COPD models and validated the use of AST in improving the pathological condition of lung tissues of mice with COPD and treating diaphragmatic injury.

### 3.2 Effect of AST on diaphragm apoptosis in COPD mice models

**FIGURE 2:**
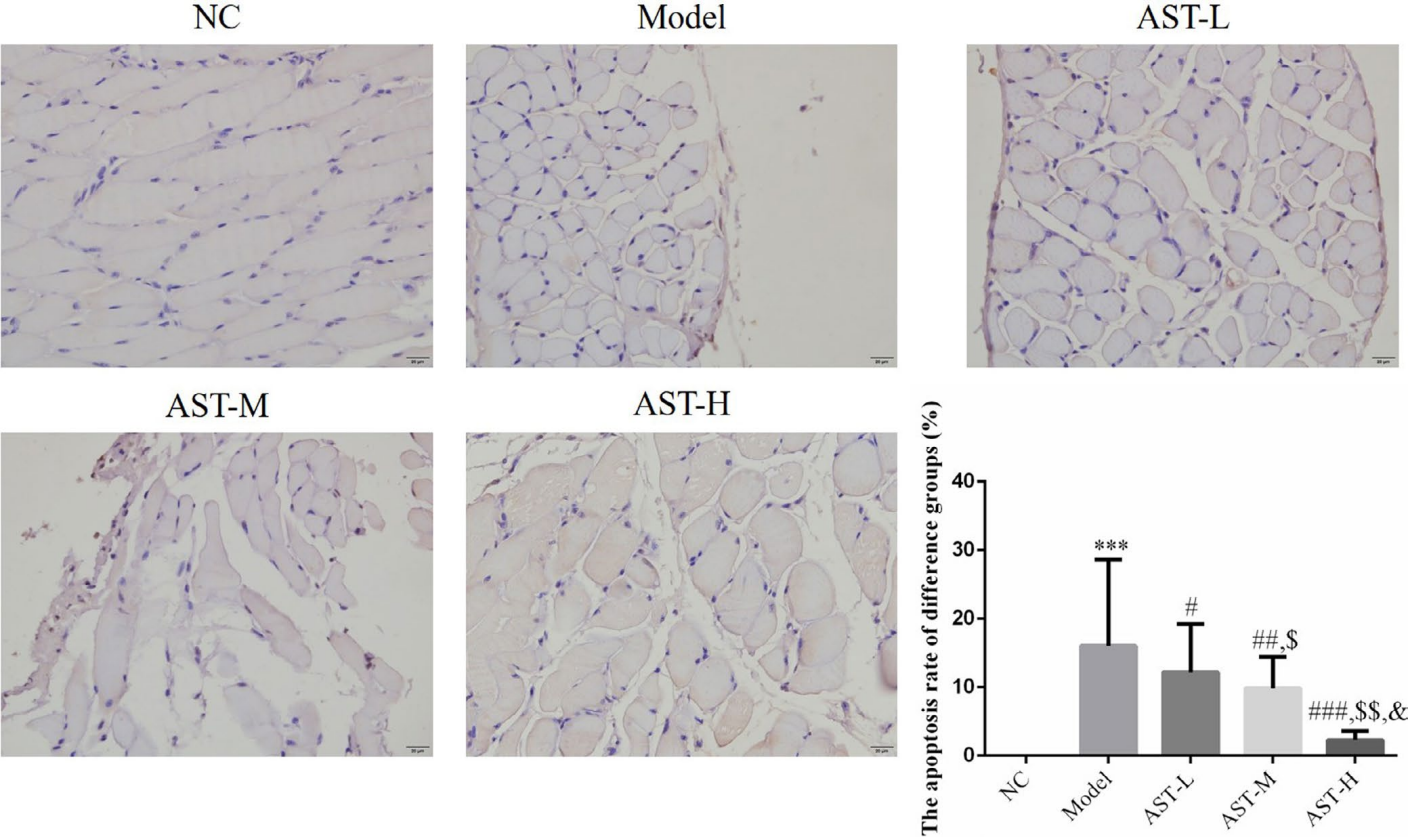
Diaphragm cell apoptosis number of difference groups by TUNEL assay. NC, mice treated with normal control; model, COPD model mice; AST‐L, COPD model mice treated with low‐dose AST; AST‐M, COPD model mice treated with middle‐dose AST; AST‐H, COPD model mice treated with high‐dose AST. ***: *p* < .001 compared with the NC group; #: *p* < .05, ##: *p* < .01, and ###: *p* < .001 compared with the model group; $: *p* < .05 and $$: *p* < .01 compared with the AST‐L group; and &: *p* < .05 compared with the AST‐M group

TUNEL staining showed that the apoptotic rate of the diaphragm cells of the model group was remarkably higher than that the NC group (*p* < .001). In the mice administered with AST, the apoptosis of diaphragm cells was inhibited significantly (*p* < .05, respectively, Figure 2). The differences in the apoptotic rates of the AST‐L, AST‐M, AST‐H, and AST + AKT inhibitor groups were statistically significant (*p* < .05, respectively, Figure 2). Therefore, a clear dose–response relationship was observed between AST and diaphragm cell apoptosis. AST at a dose of as high as 20 mg/kg·d showed the optimal efficacy in reducing apoptotic cells in the diaphragm.

### 3.3 IHC analysis of the effect of AST on relevant proteins

**FIGURE 3:**
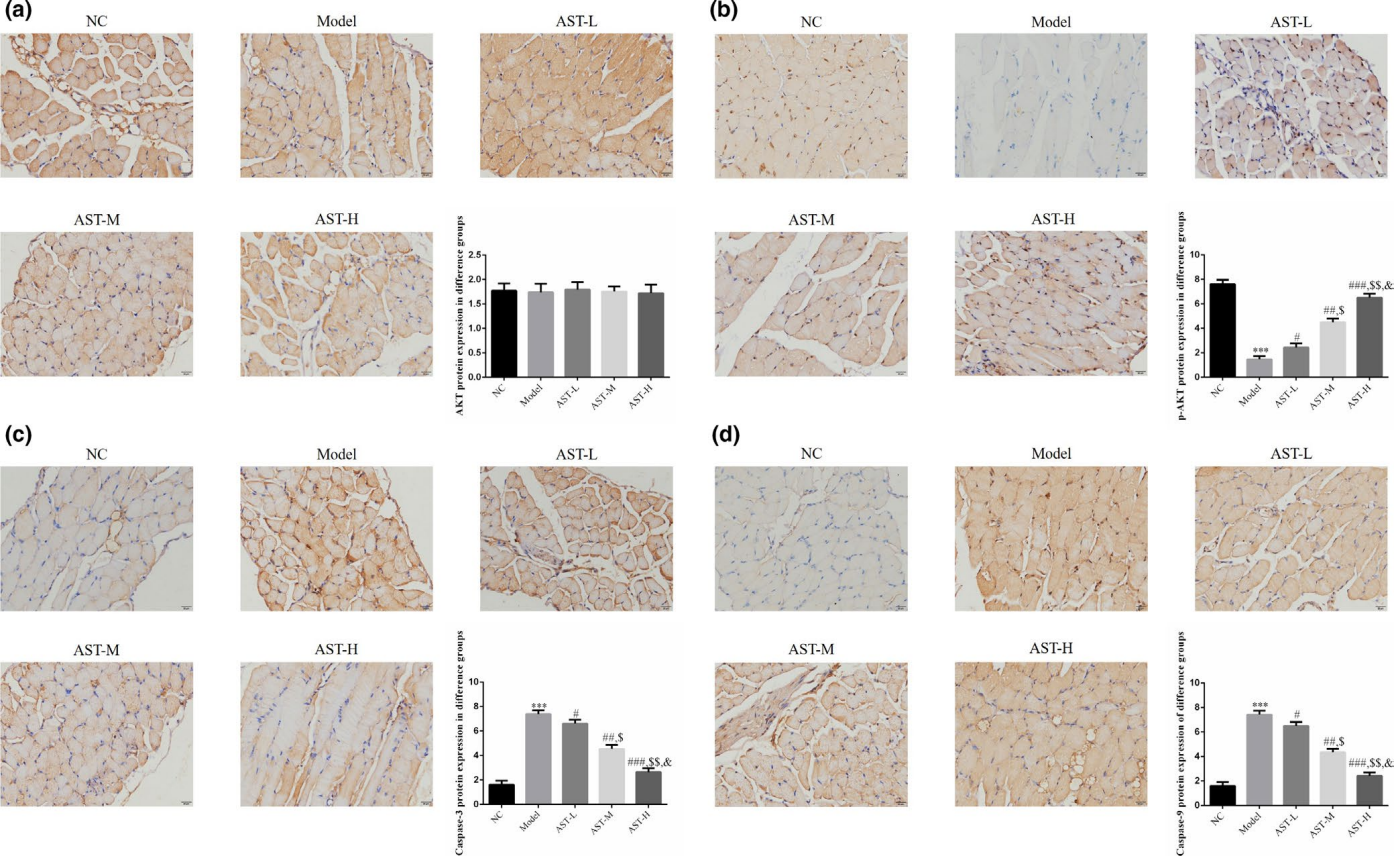
IHC assay of the relative protein expression in the diaphragm tissues. NC, mice treated with normal control; model, COPD model mice; AST‐L, COPD model mice treated with low‐dose AST; AST‐M, COPD model mice treated with middle‐dose AST; AST‐H, COPD model mice treated with high‐dose AST. (a) IHC assay (400×) of the AKT protein expression in the diaphragm tissues of different groups. (b) IHC assay (400×) of the p‐AKT protein expression in the diaphragm tissues of different groups. (c) IHC assay (400×) of the caspase‐3 protein expression in the diaphragm tissues of different groups. (d) IHC assay (400×) of the caspase‐9 protein expression in the diaphragm tissues of different groups. ***: *p* < .001 compared with the NC group; #: *p* < .05, ##: *p* < .01, and ###: *p* < .001 compared with the model group; $: *p* < .05 and $$: *p* < .01 compared with the AST‐L group; and &: *p* < .05 compared with the AST‐M group

Immunohistochemistry results indicated that the six groups show no statistically significant difference in AKT expression (*p* > .05, Figure 3a). The p‐AKT expression level in the model group decreased compared with that in the NC group, whereas the caspase‐3 and caspase‐9 expression levels significantly increased (*p* < .001, respectively, Figure 3b–d). In the AST groups, the p‐AKT expression level was elevated remarkably, whereas the caspase‐3 and caspase‐9 expression levels were reduced substantially (*p* < .05, respectively, Figure 3b–d). These results suggested a dose–response relationship.

### 3.4 WB analysis of the effect of AST on relevant proteins

**FIGURE 4:**
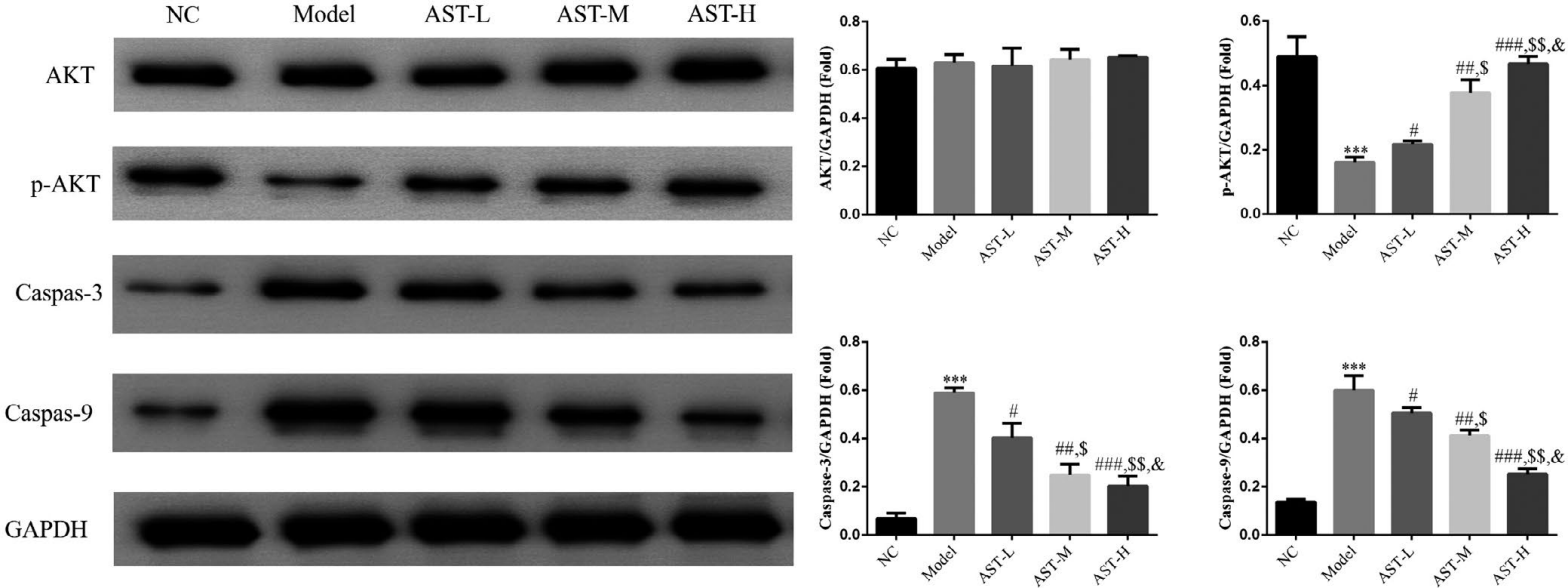
WB assay of the relative protein expression in the diaphragm tissues. NC, mice treated with normal control; model, COPD model mice; AST‐L, COPD model mice treated with low‐dose AST; AST‐M, COPD model mice treated with middle‐dose AST; AST‐H, COPD model mice treated with high‐dose AST. ***: *p < *.001, compared with NC group; #: *p < *.05, ##: *p < *.01, ###: *p < *.001, compared with Model group; $: *p < *.05, $$: *p < *.01, compared with AST‐L group; &: *p < *.05, compared with AST‐M group

The WB results indicated no significant differences in the AKT expression of the six groups (*p* > .05, Figure 4a). The p‐AKT expression level of the model group was considerably lower than that of the NC group, and the caspase‐3 and caspase‐9 expression levels highly increased in the latter compared with those in the former (*p* < .001, respectively, Figure 4b–d). The p‐AKT expression level in the AST groups was significantly elevated compared with that in the model group, whereas the caspase‐3 and caspase‐9 expression levels in the former were markedly decreased compared with that in the latter (*p* < .05, respectively, Figure 4b–d). These results indicated a dose–response relationship.

### 3.5 Effect of AKT inhibitors on the pathological characteristics of lung and diaphragm tissues of mice with AST‐treated COPD

**FIGURE 5:**
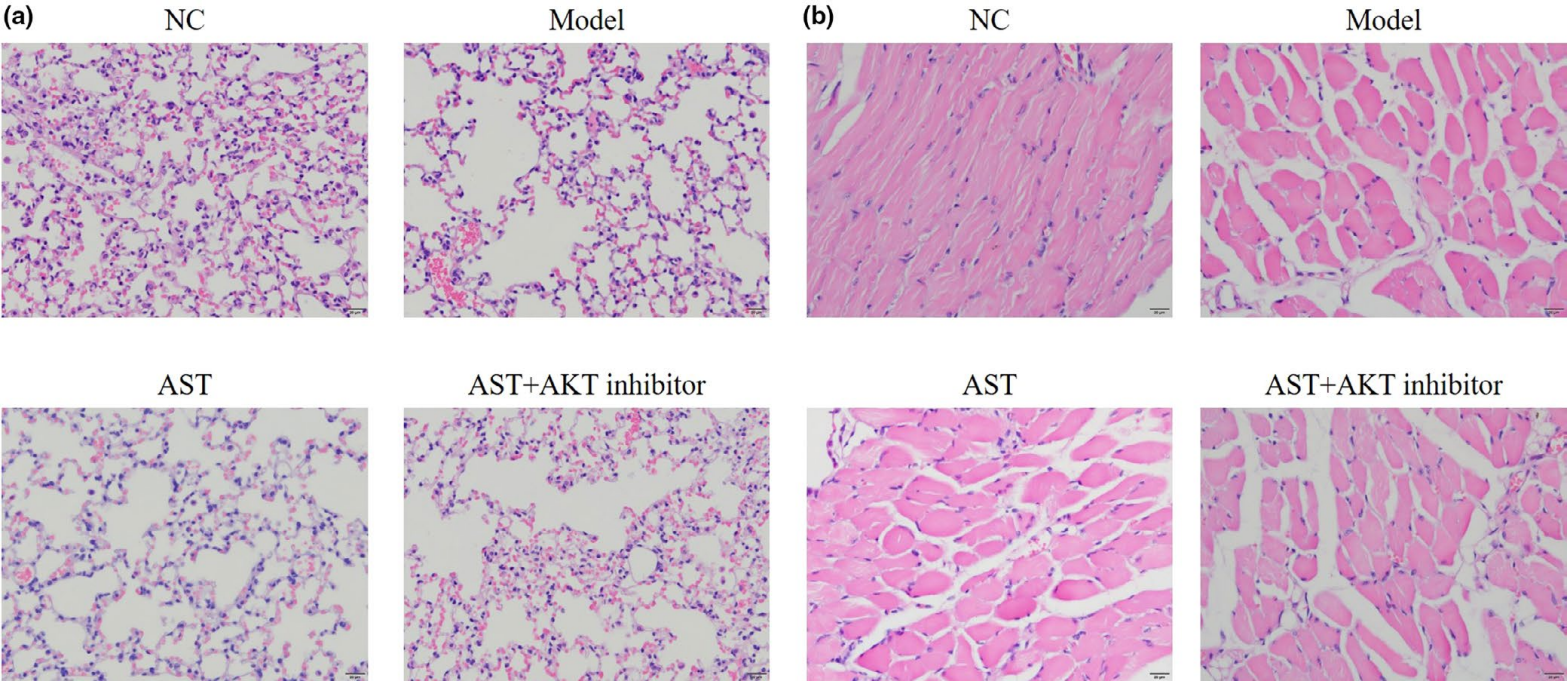
Effect of AKT inhibitors on the pathological characteristics of the lung and diaphragm tissues of mice with AST‐treated COPD. NC, mice treated with normal control; model, COPD model mice; AST, COPD model mice treated with high‐dose AST; AST+AKT inhibitor, COPD model mice treated with high‐dose AST and AKT inhibitor. (a) Pathological observation of lung tissues via HE staining (400×). (b) Pathological observation of the diaphragm via HE staining (400×)

The mice in the AST + AKT inhibitor group were administered with AST and an AKT inhibitor to further investigate the underlying mechanism on how AST improved COPD. H and E staining was performed to observe pathological changes in the lung and diaphragm tissues. Figure 5a shows that AST could effectively improve lung tissue injury, and the use of the AKT inhibitor dramatically impaired the efficacy of AST. Figure 5b indicates that AST significantly alleviated the diaphragmatic injury in mice, yet the AKT inhibitor adversely affected the outcome of AST treatment. Figure 5 presents the relative data.

### 3.6 Effect of AKT inhibitors on diaphragm cell apoptosis in AST‐treated COPD mouse models

**FIGURE 6:**
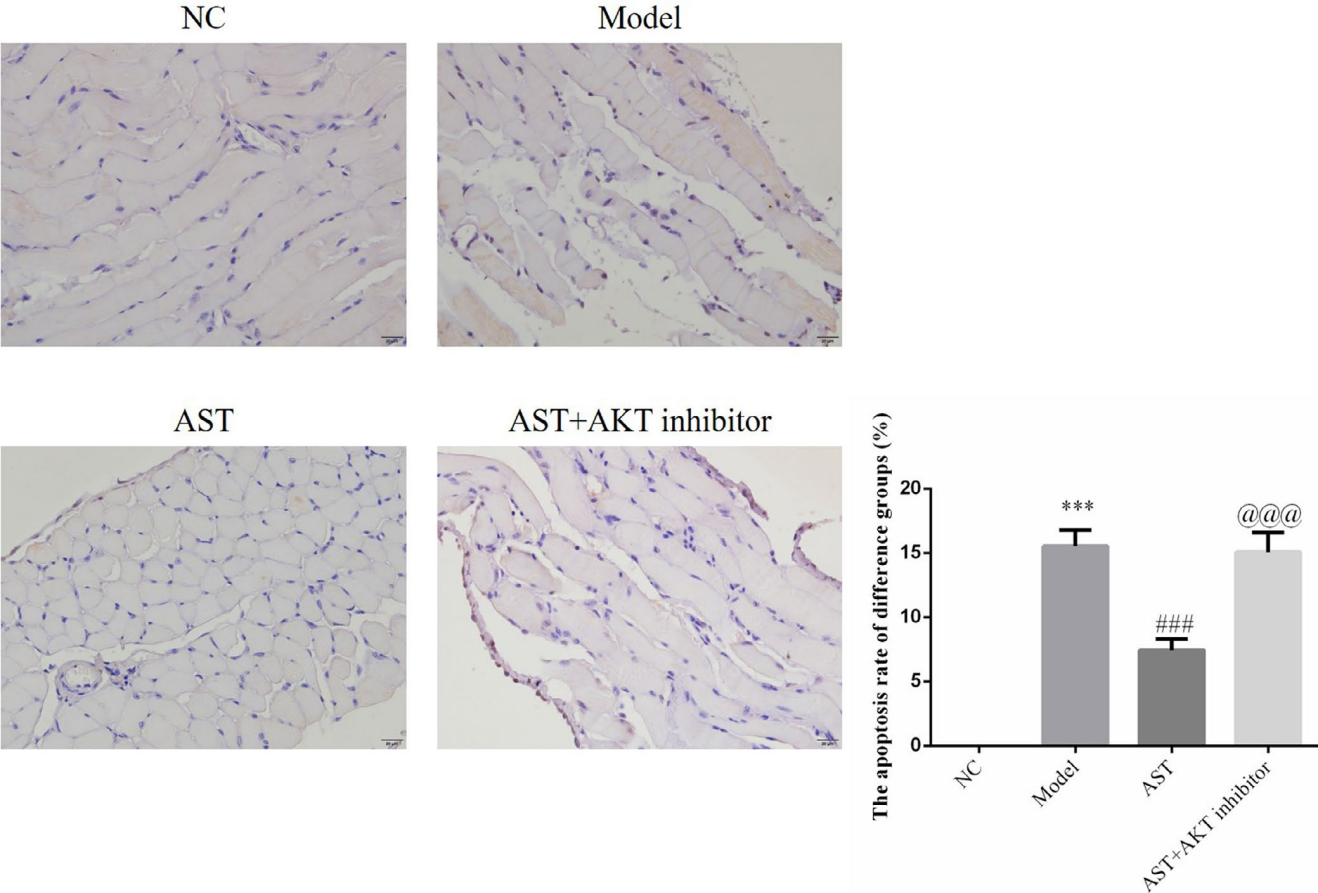
Effect of AKT inhibitors on the apoptosis of the diaphragm of mouse models with AST‐treated COPD. NC, Mice treated with normal control; Model, COPD model mice; AST, COPD model mice treated with high‐dose AST; AST + AKT inhibitor, COPD model mice treated with high‐dose AST and AKT inhibitor. ***: *p < *.001, compared with NC group; ###: *p < *.001, compared with Model group; @@@: *p < *.001, compared with AST group

The TUNEL staining results showed that the apoptotic rate of the model group was significantly increased compared with that of the NC group (*p* < .001, Figure 6). The apoptotic rates of the AST groups were much lower than that of the model group (*p* < .001, Figure 6). Unlike other AST groups, the AST + AKT inhibitor group had a dramatically increased apoptotic rate (*p* < .001, respectively, Figure 6).

### 3.7 IHC analysis of the effect of AKT inhibitors on relevant proteins

**FIGURE 7:**
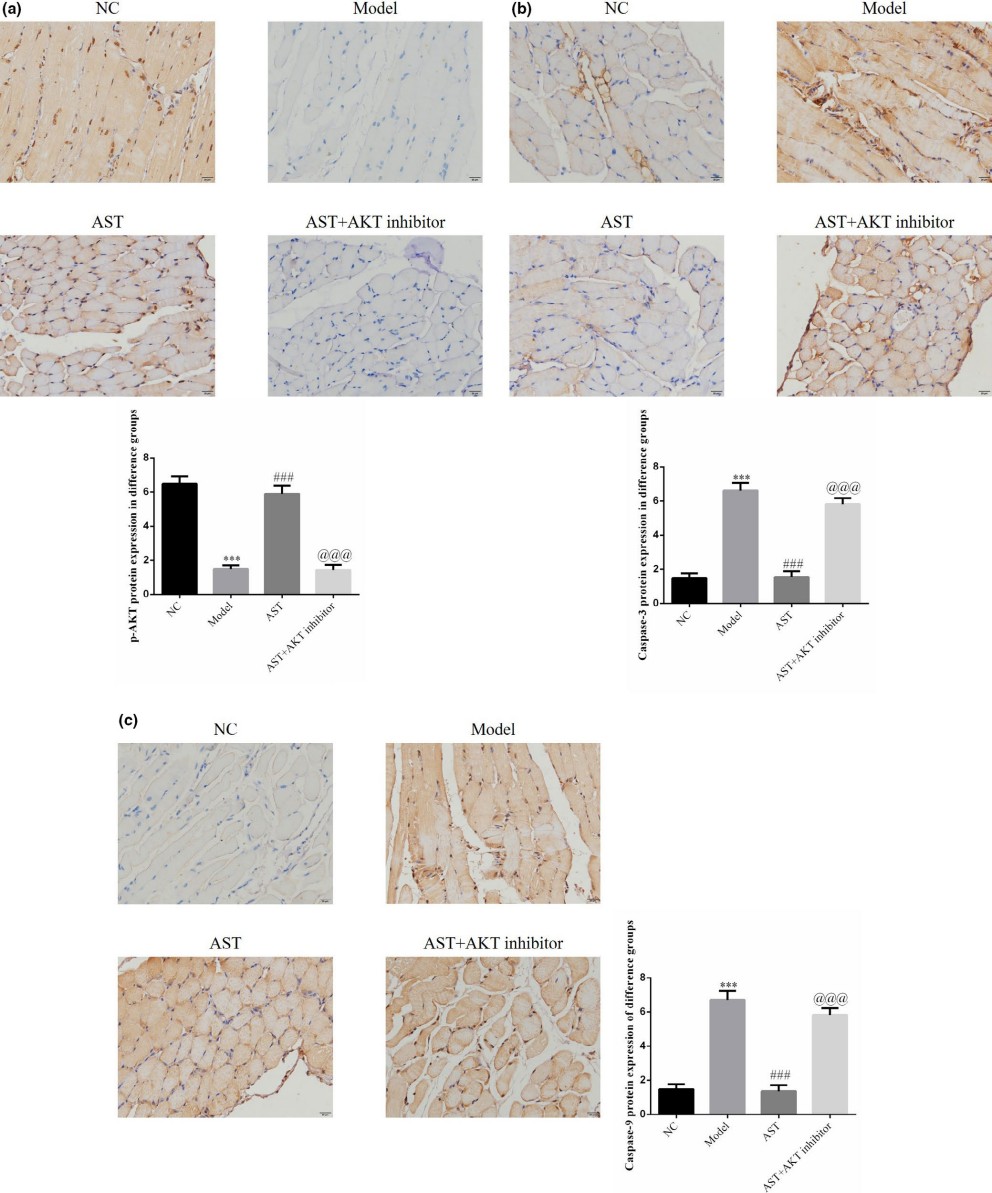
IHC assay of p‐AKT, caspase‐3, and caspase‐9 protein expressions in diaphragm tissues. NC, Mice treated with normal control; Model, COPD model mice; AST, COPD model mice treated with high‐dose AST; AST + AKT inhibitor, COPD model mice treated with high‐dose AST and AKT inhibitor. (a) IHC assay of the p‐AKT protein expression in diaphragm tissues. (b) IHC assay of the caspase‐3 protein expression in diaphragm tissues. (c) IHC assay of the caspase‐9 protein expression in diaphragm tissues. ***: *p < *.001, compared with NC group; ###: *p < *.001, compared with Model group; @@@: *p < *.001, compared with AST group

The p‐AKT expression in the model group was remarkably reduced compared with that in the NC group, whereas the caspase‐3 and caspase‐9 expression levels in the former were elevated significantly compared with that in the latter (*p* < .001, respectively, Figure 7a–c). In the AST groups, the p‐AKT expression level substantially increased, whereas the caspase‐3 and caspase‐9 expression levels dramatically decreased (*p* < .001, respectively, Figure 7a–c). When AST and AKT inhibitor were administered simultaneously, the p‐AKT expression level was reduced greatly; conversely, the caspase‐3 and caspase‐9 expression levels increased remarkably compared with that in the AST groups (*p* < .001, respectively, Figure 7a–c).

### 3.8 WB analysis of the effect of AKT inhibitors on relevant proteins

**FIGURE 8:**
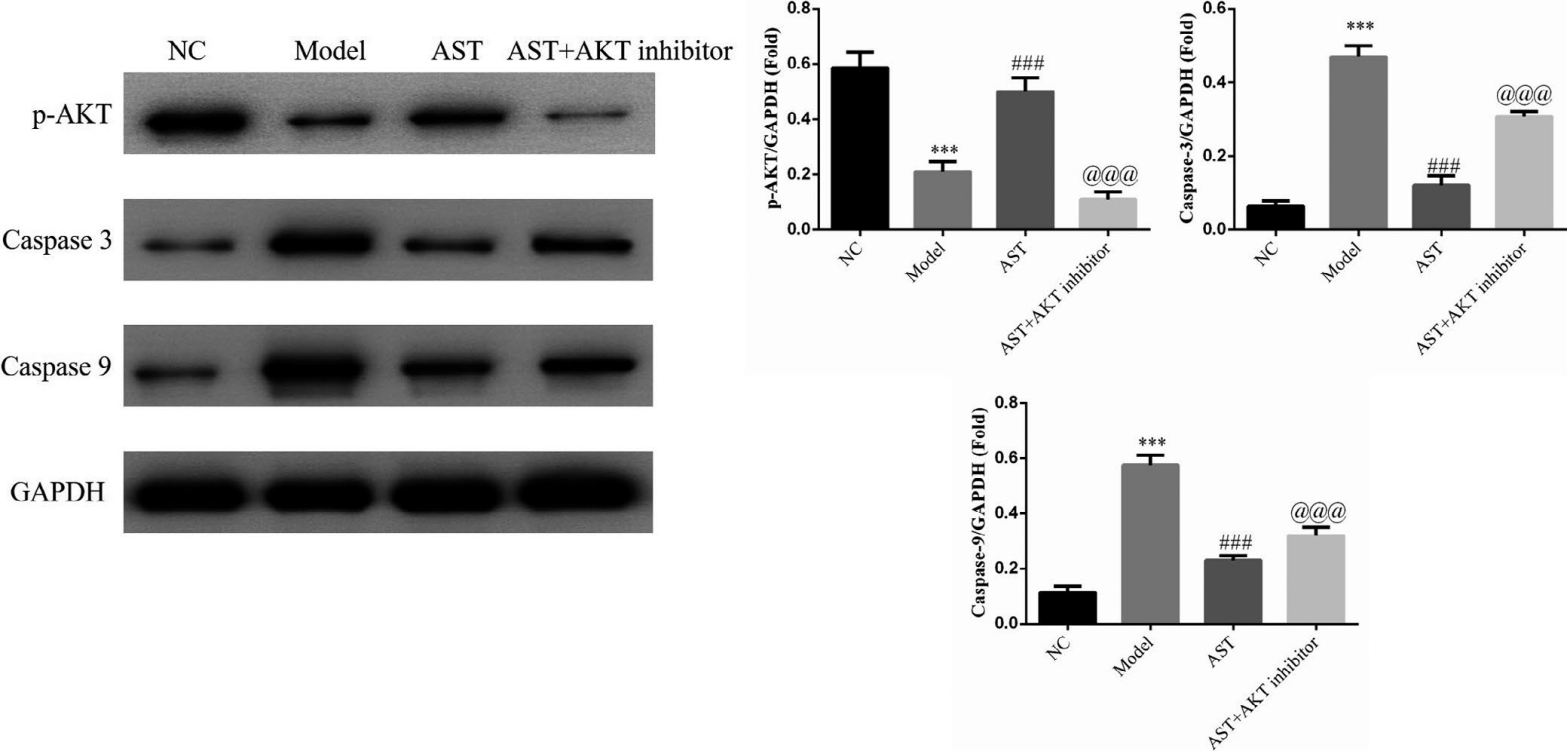
p‐AKT, caspase‐3 and caspase‐9 proteins expressions by WB assay. NC, mice treated with normal control; model, COPD model mice; AST, COPD model mice treated with high‐dose AST; AST + AKT inhibitor, COPD model mice treated with high‐dose AST and AKT inhibitor. ***: *p < *.001, compared with NC group; ###: *p < *.001, compared with Model group; @@@: *p < *.001, compared with AST group

The p‐AKT expression level in the model group significantly decreased, whereas the caspase‐3 and caspase‐9 expression levels markedly increased compared with those in the NC group (*p* < .001, respectively, Figure 8a–c). The p‐AKT expression level in the AST groups markedly increased compared with that in the model group, whereas the caspase‐3 and caspase‐9 expression levels in the former were dramatically reduced compared with that in the latter (*p* < .001, respectively, Figure 8a–c). The p‐Akt expression level in the AST + AKT inhibitor group was decreased remarkably compared with that in the AST groups; conversely, the caspase‐3 and caspase‐9 expression levels in the former were substantially increased compared with that in the latter (*p* < .001, respectively, Figure 8a–c).

## 4 DISCUSSION

The incidence of COPD is associated with the chronic inflammatory responses of the airways and the lungs to toxic and harmful gases or particles. COPD has become a serious threat to public health because it can induce chronic systemic inflammation and has increasing incidence, disability, and fatality rates. A risk factor of COPD is cigarette smoking. About 90% of patients with COPD are smokers. Cigarette smoke contains multiple toxic gases and other substances that may lead to the development of COPD (Ferhani et al., 2010). Patients with COPD and a smoking history, even those who have quitted smoking for years, may present with inflammatory responses of the lungs; in this case, different types of inflammatory cells significantly outnumber those in healthy controls. Most patients with COPD have skeletal muscle atrophy. The diaphragm is a skeletal muscle and the most important respiratory muscle. In the case of COPD, the diaphragm is heavily loaded and shows a functional disadvantage in oxygen delivery, often leading to muscle atrophy at varying degrees and reduced diaphragmatic contractility (Supinski, Vanags, & Callahan, 2009; Zhang et al., 2020). If the diaphragm muscle atrophies, patients with COPD may suffer from respiratory failure and have a poor prognosis as this disease worsens. In the present study, COPD mouse models were well established using cigarette smoke. The mouse models revealed that the apoptotic rate of the COPD group was higher than that of the NC group. This finding suggested that the components of cigarette smoke could induce the apoptosis of diaphragm muscle cells.

As a typical traditional Chinese medicine, AST has been widely used for anti‐inflammatory treatment (Hsieh, Liu, Chen, Huang, & Wu, 2020; Xie et al., 2020; Zhang et al., 2020). Our study indicated that AST was an effective solution to the COPD‐induced pathological changes in the lung and diaphragm tissues. The TUNEL assay showed that AST could effectively inhibit COPD‐induced diaphragm cell apoptosis.

Apoptosis pathways are diverse. Caspases play an essential role in apoptosis, particularly in relevant physiological and pathological processes. As such, caspases are common pathways transmitting multiple apoptotic signals (Xing, Wang, Su, Cui, & Li, 2012). Caspases exist as inactive zymogens in the cytoplasm of normal cells. When caspases are activated, a cascade is triggered to produce hydrolyzed proteins and stimulate irreversible apoptosis. Multiple signal transmitting pathways act collectively with caspases to induce apoptosis. Particularly, caspase‐9 is an initiator caspase participating in mitochondrial apoptosis and other apoptosis pathways (Ijiri et al., 2018). Caspase‐3 is one of the major executioners of apoptosis (Langford, McGee, Ta, Redens, & Texada, 2011). In the present study, the expression levels of caspase‐9 and caspase‐3 in diaphragm tissues were significantly higher in the model group than in the NC group. This result indicated that diaphragm apoptosis in the mice with COPD was strongly associated with the activation of caspases and relevant cascade reactions. After the AST treatment, the caspase‐9 and caspase‐3 expression levels markedly decreased, whereas the p‐AKT expression level sharply increased.

AKT is a clinically common serine/threonine‐specific protein kinase, and its phosphorylated form is p‐AKT, which shows biological activity. With homology to protein kinases A and C, AKT is also known as protein kinase B (PKB) (Ma et al., 2020; Wang et al., 2020). p‐Akt is a cell cycle regulator, proliferation promoter, and apoptosis inhibitor by acting on its downstream molecules. In the present study, some mice with COPD were simultaneously treated with AST and AKT inhibitor. In this case, the efficacy was offset when the AKT inhibitor was added. Therefore, AST might inhibit the expression of caspase‐9 and caspase‐3 by activating p‐AKT, thereby producing favorable therapeutic outcomes in COPD cases.

In conclusion, the in vivo mouse models demonstrated that AST could inhibit caspase‐9 and caspase‐3 expression by activating p‐AKT to help improve COPD‐induced diaphragm apoptosis, alleviate diaphragmatic fatigue, and reduce the risk of respiratory failure.
